# Development of a Prediction Model for Demolition Waste Generation Using a Random Forest Algorithm Based on Small DataSets

**DOI:** 10.3390/ijerph17196997

**Published:** 2020-09-24

**Authors:** Gi-Wook Cha, Hyeun Jun Moon, Young-Min Kim, Won-Hwa Hong, Jung-Ha Hwang, Won-Jun Park, Young-Chan Kim

**Affiliations:** 1 Department of Architectural Engineering, Dankook University, Yongin 16890, Korea; cgwgnr@gmail.com (G.-W.C.); hmoon@dankook.ac.kr (H.J.M.); 2 Department of Applied Statistics, Dankook University, Yongin 16890, Korea; dudals4051@gmail.com; 3 School of Architecture, Civil, Environmental and Energy Engineering, Kyungpook National University, Daegu 41566, Korea; hongwonhwa@gmail.com; 4 School of Architecture, Kyungpook National University, Daegu 41566, Korea; peter@knu.ac.kr; 5 Department of Architectural Engineering, Kangwon National University, Gangwon-do 25913, Korea; 6 Department of Fire and Disaster Prevention Engineering, Changshin University, Gyeongsangnam-do 51352, Korea

**Keywords:** demolition waste management, construction waste management, prediction model, random forest, leave-one-out cross-validation, small data

## Abstract

Recently, artificial intelligence (AI) technologies have been employed to predict construction and demolition (C&D) waste generation. However, most studies have used machine learning models with continuous data input variables, applying algorithms, such as artificial neural networks, adaptive neuro-fuzzy inference systems, support vector machines, linear regression analysis, decision trees, and genetic algorithms. Therefore, machine learning algorithms may not perform as well when applied to categorical data. This article uses machine learning algorithms to predict C&D waste generation from a dataset, as a way to improve the accuracy of waste management in C&D facilities. These datasets include categorical (e.g., region, building structure, building use, wall material, and roofing material), and continuous data (particularly, gloss floor area), and a random forest (RF) algorithm was used. Results indicate that RF is an adequate machine learning algorithm for a small dataset consisting of categorical data, and even with a small dataset, an adequate prediction model can be developed. Despite the small dataset, the predictive performance according to the demolition waste (DW) type was R (Pearson’s correlation coefficient) = 0.691–0.871, R^2^ (coefficient of determination) = 0.554–0.800, showing stable prediction performance. High prediction performance was observed using three (for mortar), five (for other DW types), or six (for concrete) input variables. This study is significant because the proposed RF model can predict DW generation using a small amount of data. Additionally, it demonstrates the possibility of applying AI to multi-purpose DW management.

## 1. Introduction

Large volumes of waste are generated from various construction and demolition (C&D) activities worldwide [1,2,3]. Accordingly, many studies in the literature have focused on the importance of optimal C&D waste management [4]. In particular, demolition waste (DW) generated from building or structure demolition has been reported to account for approximately 70–90% of the total C&D waste [5,6]. Building demolition in cities is required for new construction because of land shortages [7]. For C&D waste management, calculating C&D waste generation has been recognized as a useful method. This is a methodology that estimates C&D waste generation using a generation rate calculation (GRC) or C&D waste generation coefficient [8]. The principle of this method is to obtain the waste generation rate (in units such as kg/m^2^ or m^3^/m^2^). Several researchers have developed methodologies to quantify C&D waste by establishing ranges and parameters [9]. For example, Oliver et al. (2010) [10] conducted a study in which the generated C&D waste was estimated (in tons and m^3^) using reliable simulation methods. Additionally, researchers, such as [11,12,13] estimated the total amount of C&D waste generated by multiplying the generation rate by the total area. As mentioned previously, the amount of waste generated was predicted using a waste generation index, such as the waste generation rate obtained by statistical analysis based on gross floor area (GFA) [14]. However, recently, artificial intelligence (AI) technologies have been increasingly employed to accurately predict C&D waste generation [15]. AI algorithms are regarded as state-of-the-art models for reliable prediction of a waste generation because of their unique features (i.e., data input, learning, and prediction) [16].

Machine learning and statistical analysis algorithms that have been employed to predict C&D waste generation include artificial neural networks (ANNs), adaptive neuro-fuzzy inference systems, support vector machines (SVMs), linear regression (LR) analysis, decision trees (DTs), and genetic algorithms (Gas) [15]. Furthermore, most existing studies on the prediction of C&D waste generation have used machine learning models based on using continuous data as input variables that applied algorithms, such as ANN (Golbaz et al. (2019) [17]; Noori et al. (2010) [18]; and Song et al. (2016) [19]), SVM (Abbasi et al. (2013) [20]; Golbaz et al. (2019) [17]; and Kumar et al. (2018) [21]), LR (Abdoli et al. (2011) [22]; Azadi and Karimijashni (2015) [23]; Chhay et al. (2018) [24]; and Golbaz et al. (2019) [17]), and DT (Cha et al. (2017) [25]; Huang et al. (2011) [26]; and Kannangara et al. (2017) [27]). However, independent variables, which include continuous data (e.g., building age, GFA) and categorical data (e.g., region [28], building use [29], building structure [30], wall material [31], and roofing material [31]), affect C&D waste generation. For instance, the type of building structure affects the choice of construction techniques [32], which in turn determines the generation and constituents of C&D waste. Accordingly, it cannot be guaranteed that the machine learning algorithms used in existing studies will perform well when applied to categorical data instead of continuous data.

Therefore, in this study, we explored a method of utilizing machine learning algorithms for a dataset that includes categorical data (region, building structure, building use, wall material, and roofing material), as well as continuous data (especially, GFA) to improve the accuracy of C&D waste generation prediction. Thus, 784 buildings reported in the existing literature [25] were examined as follows:

(1) Deduction of a machine learning algorithm that fits the data features—Random forest (RF) was found to be an adequate machine learning algorithm given the features of the data used in this study after theoretical considerations;

(2) Selection of input variables to improve model performance—Data preprocessing was performed to normalize and remove outliers from the raw data to improve model performance using the RF algorithm. Then, feature selection was performed using RF-recursive feature elimination (REF) to identify important input variables affecting the prediction outcomes by the DW type;

(3) Deduction of a general prediction model by machine learning for each DW type—Prediction models for each DW type (11 models) and a prediction model for individual buildings (one model) were derived;

(4) Verification and evaluation of prediction models—The RF models were verified by leave-one-out cross-validation (LOOCV), whereas the predictive model performances were evaluated by R (correlation coefficient) and R^2^ (coefficient of determination) values, which are performance indicators representing the correlation between observed and predicted values. For the machine learning data, the amount of DW generated (kg/m^2^) by the DW type (mortar, concrete, block, brick, timber, slate, roofing tile, plastic, glass, metal, and soil) was used as suggested by Cha et al. [25].

Consequently, we demonstrated that prediction models’ results can provide more reliable predictions than existing machine learning methods when the RF algorithm with categorical variables is applied. This study is highly significant in that it proposed an RF model that can predict DW generation using a relatively small amount of data. However, this study used limited data. In future studies, the data range should be extended, and comparative analysis should be performed using various machine learning algorithms. This study is also meaningful because it emphasized the possibility of applying AI to multi-purpose DW management.

## 2. Materials and Methods

We used existing raw data (shown in Cha et al. [25]) from 784 buildings for the generated amounts (kg) of 11 DW types (mortar, concrete, block, brick, timber, slate, roofing tile, plastic, glass, metal, and soil) for six input variables (GFA, region, building structure, building use, wall material, and roofing material). Because the independent variables affecting model outcome were mostly categorical data, it was critical to select the appropriate machine learning algorithm. According to Cha et al. [25], a statistical model using the DT algorithm can produce excellent predictions for DW generation. However, this model did not use machine learning and was developed based on statistical results. Given the characteristics of DT algorithms, there is a strong likelihood that the model will be generated based on highly influential variables. It also may exhibit bias [33] and high variance [34] because a small dataset was used rather than a large dataset. Therefore, in this study, we focused on machine learning models for DW generation prediction to avoid such limitations and achieve stable prediction performance.

For the data unit of DW generation, DW generated (kg/m^2^) per GFA (m^2^) was used in modeling, which is given by the following equation:

(1) $${\mathrm{DWGR}}_{i}\;\mathrm{of}\;\mathrm{building}=\frac{\sum A_{ij}\;\mathrm{of}\;\mathrm{building}}{\mathrm{GFA}\;\mathrm{of}\;\mathrm{building}}$$

where DWGR is the demolition waste generation rate (kg/m^2^), $A_{ij}$ is the amount of material *j* with properties of waste material I (quantity) (kg), and GFA is the gross floor area (m^2^).

### 2.1. Random Forest Algorithm

**Figure 1:**
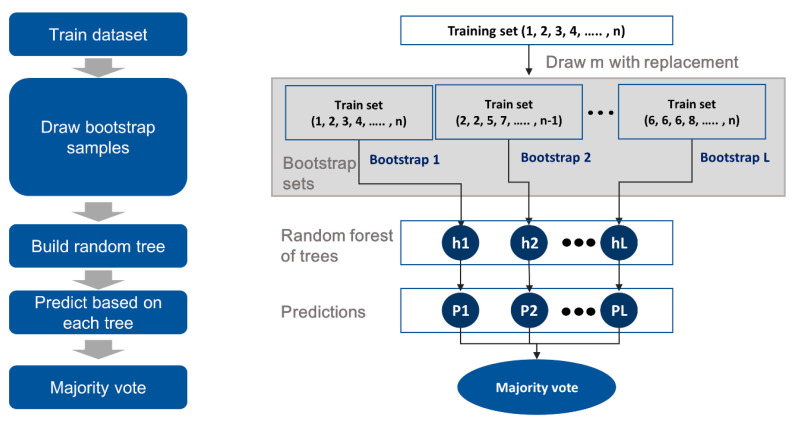
Structure of the random forest (RF) algorithm.

Random forest (RF) is an ensemble classifier that uses multiple models containing several DTs to obtain a better prediction performance. It creates many classification trees, and a bootstrap sample technique is used to train each tree from the set of training data. This method only searches for a random subset of variables in order to obtain a split at each node. For classification, the input vector is fed to each tree in the RF, and each tree votes for a class. Finally, the RF chooses the class with the highest number of votes, as shown in Figure 1.

The RF algorithm can be used to address the above-mentioned problems of the DT algorithm. It consists of multiple DTs and is a machine learning model where the DT model forms an ensemble with bagging (an approach using a set of base models) [35]. Hence, the RF algorithm can reduce data variance and prevent the strong dependence of the DT model on highly influential variables. Bagging is an ensemble method that uses bootstrap sampling. It randomly selects a sample size of n from a subset of data and chooses m input variables arbitrarily to generate a DT model. By repeating the generation process, it produces the final result by voting of the generated DT models. Thus, the RF algorithm can avoid overfitting and the influence of outliers [36]. Even when a dataset has unbalanced classes, RF can produce more accurate predictions than other algorithms [37,38]. Thus, we chose the RF algorithm considering that it would provide more reliable predictions than the previously reported machine learning algorithms [25].

### 2.2. Feature Selection Methods

To improve the prediction performance of the model, input variables that have low correlations are generally excluded from modeling, leaving out influential or highly influential variables [39]. There are three representative methods for input variable selection: The filter method, wrapper method, and embedded method [40]. First, the filter method provides a ranking of input variables against output variables independently from machine learning algorithms [40]. Second, the wrapper method ranks the input variables after establishing a machine learning model. A considerable amount of time is required for calculations on all input variables; however, it enables accurate variable selection. Lastly, the embedded method employs different application methods depending on the machine learning algorithm [40]. It is used when selecting variables in the training process for machine learning. The representative embedded methods are SVM-RFE (recursive feature elimination) and RF-RFE [41]. These RFE methods include all variables in the process and eliminate less significant variables one-by-one, while repeating the learning process. They are applicable even without assuming a normal data distribution and exhibit higher performance in variable selection and prediction when the difference in significance between the variables is large [36]. Thus, because of the two reasons presented above (i.e., the data used in this study does not follow a normal distribution, and the RF algorithm is applied), the RF-RFE method was used for input variable selection in this study.

### 2.3. Leave-One-Out Cross-Validation

**Figure 2:**
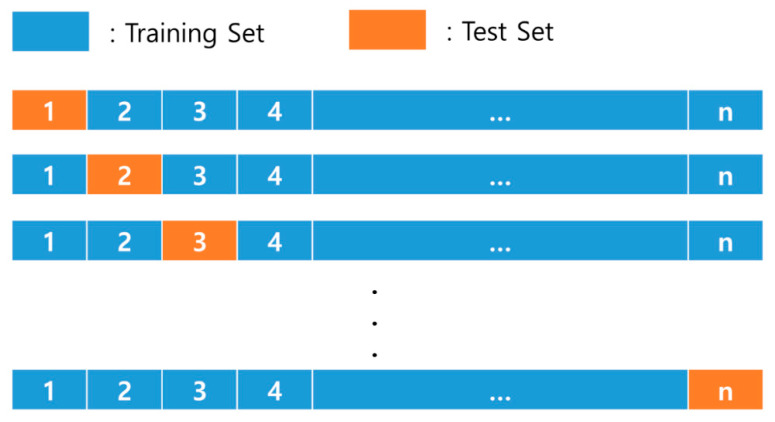
Schematic representation of the leave-one-out cross-validation (LOOCV) method.

Leave-one-out cross-validation (LOOCV) is useful when evaluating the performance of machine learning when a dataset or category value is small [42]. LOOCV takes one data sample from n data samples as a test set and the remaining n−1 data samples as a training set to evaluate model performance. Because it uses only one sample as a test set, models can be generated using n training data. It also provides relatively stable results by testing all samples when compared to the validation set approach, which is the existing cross-validation method (10-fold or k-fold). The LOOCV is a nearly unbiased and reliable method of estimating the performance of a machine learning model as long as the training and testing sets are drawn from the same distribution [43]. The DW data of 784 buildings used in this study form a rather small dataset. Hence, the existing validation method could cause serious bias and overestimation [33]. Therefore, this study used the LOOCV method to develop a stable RF prediction model. Figure 2 shows a schematic representation of the LOOCV method.

### 2.4. Limitations of LOOCV and the Results of the RF Model in this Study

LOOCV is an extreme version of k-fold cross-validation that has the maximum computational cost. It requires one model to be created and evaluated for each example in the training dataset. The benefit of using so many fitted and evaluated models is a more robust estimate of model performance as each row of data is given an opportunity to represent the entirety of the test dataset. However, it is a computationally expensive procedure to perform, although it results in a reliable and unbiased estimate of model performance. Although it is simple to use and there is no configuration to specify, there are times when the procedure should not be used, such as in the case of the evaluation of a significantly large dataset or a computationally expensive model [43]. This study utilized the DW dataset for 784 buildings. However, considering the fact that AI models are mainly driven by extensive datasets [15], the DW RF model developed in this study may suffer from a lack or incompleteness of waste data.

## 3. Development of a DW Prediction Model

### 3.1. Overview of the RF Model Development Method for Predicting DW Generation

**Figure 3:**
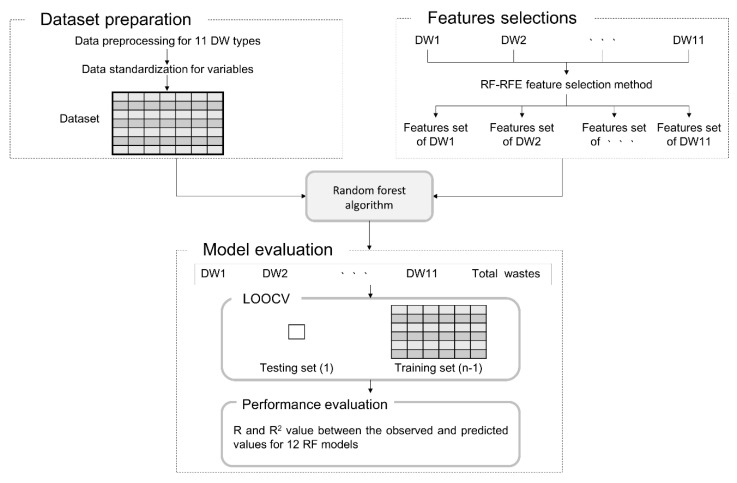
The methodology of the RF model developed in this study.

In this section, an RF modeling method for predicting DW generation, as shown in Figure 3, is presented. In this study, a dataset to be applied to the RF model was constructed based on the existing raw data (shown in Cha et al. (2017) [25]). As shown in Figure 3, dataset construction and preparation were performed through data preprocessing, and outliers were removed from the input variables for 11 DW types. Standardization was performed on the area of the building (GFA), the only continuous variable among the input variables (i.e., GFA, region, building structure, building use, wall material, and roofing material). However, the other variables were nominal variables that were not included in the standardization process. Table 1 lists the characteristics and composition of the variables applied to the RF model. The RF-RFE was applied as a feature selection method to derive important variables that affect the results from modeling, and the derived feature set based on this was used as an input variable to generate an RF model for each DW type. Finally, the LOOCV method was applied to verify the performance of RF models, and the predictive performance of RF models was determined using R (Pearson’s correlation coefficient) and R^2^ (coefficient of determination).

### 3.2. Data Preprocessing and Input Variable Selection

To improve the performance of the RF model, it is important to construct a stable dataset and select the right input variables. Thus, data preprocessing was conducted to remove outliers from raw data, and data standardization was performed to enhance the performance of the prediction model. Prior to data preprocessing, outliers were first removed by an outlier detection method using the interquartile range (IQR) (1Q (Quartile) − 1.5 × IQR (Q3 – Q1) (interquartile range) < selecting data < 3Q + 1.5 × IQR). Input data standardization was conducted using Equation (2), in which the average of the data is $x_{average}$, and the standard deviation is $\sigma_{standard\;deviation}$. In this study, we developed an RF algorithm model using a dataset of 691 data selected from the raw data for 784 buildings.

(2) $$x_{standardization}=\frac{x_{element}-x_{average}}{\sigma_{standard\;deviation}}$$

To further improve the RF model, we used only highly influential variables as input variables, excluding those with low influence. The method mentioned in Section 2.2 was used to select input variables. The RF-RFE method calculated the accuracy scores for each case and provided a variable set that records the highest score as a character string as the output. The input variables that were favorable for the RF model were included in the variable set. Variable composition and number were determined considering the root mean square error, the coefficient of determination (R^2^), and the mean absolute error. The input variables for 11 DW types were deduced as a result.

### 3.3. Model Verification and Performance Evaluation

The LOOCV method mentioned in Section 2.3 was used to verify the outcome prediction model developed in this study. As a distinct indicator for evaluating the performance of a machine learning model, R (Pearson’s correlation coefficient) or R^2^ (coefficient of determination) are useful. Thus, many researchers (e.g., Qi et al. (2018) [44]; Han et al. (2020) [45]; Kannangara et al. (2018) [27]; Kumar et al. (2018) [21]) used the R or R^2^ value between the observed and predicted value for assessing the performance of prediction models. In this study, for performance evaluation of the prediction model, the R (correlation coefficient) value, which is given by Equations (3) and (4), between the observed and predicted values was used as the performance indicator.

(3) $$R=\frac{\sum_{i=1}^{n}\left(Y_{i}-\bar{Y}\right)\left(\overset{⏞}{Y_{i}}-\bar{\overset{⏞}{Y}}\right)}{\sqrt{\sum_{i=1}^{n}\left(Y_{i}-\bar{Y}\right)\sum_{i=1}^{n}\left(\overset{⏞}{Y_{i}}-\bar{\overset{⏞}{Y}}\right)}}$$

(4) $$R^{2}=1-\frac{\sum_{i=1}^{n}{\left(\overset{⏞}{Y_{i}}-Y_{i}\right)}^{2}}{\sum_{i=1}^{n}{\left(Y_{i}-\bar{Y}\right)}^{2}}$$

where $Y_{i}$ is the observed value of the generated DW amount, $\overset{⏞}{Y_{i}}$ is the predicted value of the generated DW amount, $\bar{Y}$ is the average observed value of the generated DW amounts, $\bar{\overset{⏞}{Y}}$ is the average predicted value of the generated DW amount, and n is the number of samples.

## 4. Results

### 4.1. Results of Input Variable Selection

As mentioned in Section 2, we used six raw data input variables and deduced input variables by the DW type for the RF model using RF-RFE, as shown in Table 1. The variables affecting the RF model outcome were different based upon the DW type, and their significance also varied by depending on the combination of input variables. Considering the influence variables by the DW type, mortar appeared to be highly affected by three input variables (structure, region, and GFA), excluding building use, roofing material, and GFA from the six variables. In other words, by applying the RF algorithm, it is possible to obtain a model for predicting the amount of mortar generation with good predictive performance with only three variables (i.e., structure, region, and GFA). In contrast, the variable set for concrete contained all six input variables. Hence, the RF model for concrete prediction will produce the best prediction when all the input variables are considered. The other DW types (block, brick, timber, slate, roofing tile, plastic, glass, and metal) had sets of five variables. Thus, their RF models will have the best prediction performance when five input variables are used. However, the results can vary depending on the combination of input variables, even when the same number of variables is used for each DW type. Therefore, in this study, we used three (for mortar), five (for block, brick, timber, slate, roofing tile, plastic, glass, metal, and soil), and six (for concrete) input variables to generate RF prediction models based on the result of the RF-RFE input variable selection method, shown in Table 2.

### 4.2. Prediction Performance of the Developed RF Model

Overall, one RF model that includes all 11 DW types and 11 RF model for each waste type was created. The RF model for DW generation prediction was developed using R Studio’s (R-Tools Technology, 250 Northern Ave, Boston, MA 02210 844-448-1212, https://rstudio.com/) ‘Random Forest’ package. Input variables, shown in Table 3, and 500 DTs were used for modeling. Since ‘Random Forest’ not only randomly selects samples, but also randomly selects columns [36], each of the 500 models can be considered to be different models. If regression is applied, the model is robust because it takes the average of the predictions from the 500 models. Additionally, if there are more than 500 models, the speed of the model is reduced; therefore, the number of DTs was selected to be 500. The performance of the developed RF models was verified by LOOCV testing and training. Based on this, the model performance was evaluated by the DW type.

**Figure 4:**
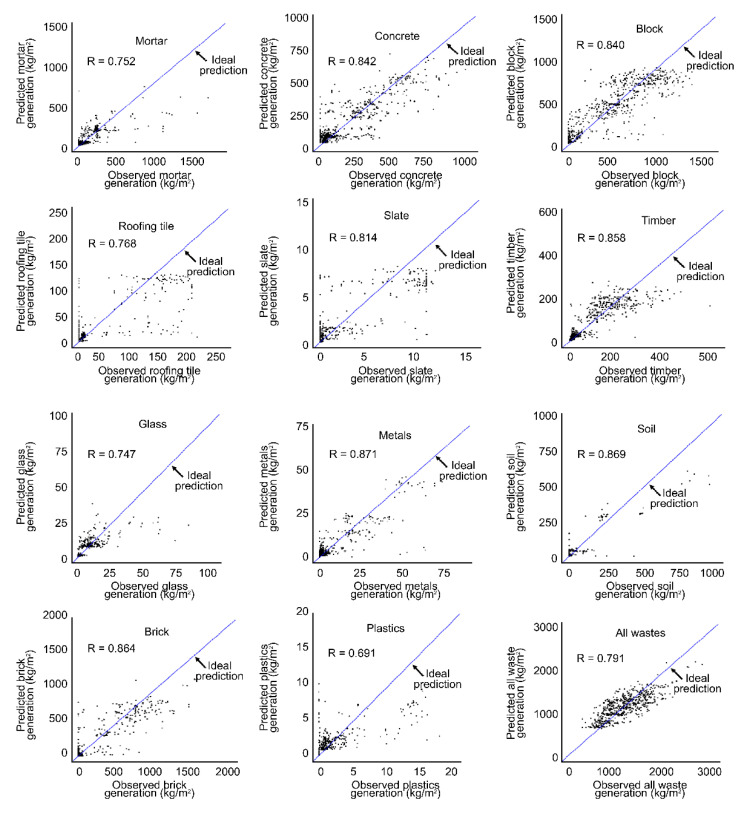
Performance of the RT model for demolition waste generation prediction.

As shown in Table 3 and Figure 4, there was a high correlation by the DW type (R = 0.691–0.871, R^2^ = 0.554–0.800) between the predicted values of the developed RF model and the observed values from the actual survey. In terms of model performance, mortar showed relatively good performance with R = 0.752. Although only three input variables (region, structure, and GFA) were used, the predictions were relatively accurate. The means of the observed and predicted values for mortar were 120.7 kg/m^2^ and 120.57 kg/m^2^, respectively, demonstrating a 0.11% error when compared. The concrete RF model also displayed high performance with R = 0.842 and R^2^ = 0.707 between the predicted values and the observed values. Given all six input variables used in the model, concrete waste generation seems to be affected by more varied input variables than the other wastes. The means of the observed and predicted values for concrete were 200.1 kg/m^2^ and 200.87 kg/m^2^, respectively, which corresponds to a 0.38% error when compared. In other models generated using the five input variables, the performance was considerably high for block (R = 0.840; R^2^ = 0.704; input variables: Wall material, region, structure, GFA, and building use; mean of observations 481.44 kg/m^2^, mean of predicted value 481.6 kg/m^2^), brick (R = 0.864; R^2^ = 0.745; input variables: Wall material, roofing material, region, GFA, and structure; mean of observations 188.6 kg/m^2^, mean of predicted value 188.1 kg/m^2^), timber (R = 0.858; R^2^ =0.735; input variables: Region, roofing material, GFA, structure, and building use; mean of observations 96.02 kg/m^2^, mean of predicted value 96.06 kg/m^2^), metal (R = 0.871; R^2^ = 0.755; input variable: Region, building use, roofing material, wall material, and structure; mean of observations 7.36 kg/m^2^, mean of predicted value 7.40 kg/m^2^), and soil (R = 0.869; R^2^ = 0.800; input variables: Region, building use, roofing material, wall material, and structure; mean of observations 20.77 kg/m^2^, mean of predicted value 20.82 kg/m^2^). From the RF model for a building that includes all DWs, the correlation between values predicted by the RF model and values observed from the actual survey was R = 0.791 and R^2^ = 0.615. The mean observed and predicted values for all wastes were 1165.04 kg/m^2^ and 1166.258 kg/m^2^, respectively, demonstrating a 0.1% error when compared.

**Figure 5:**
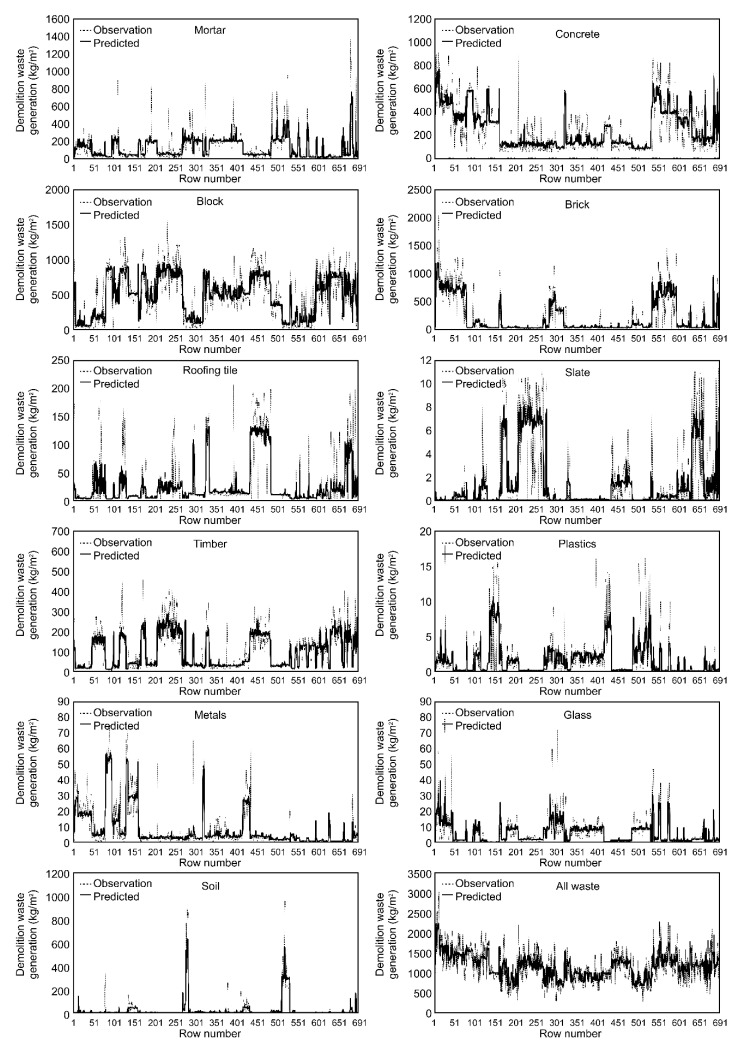
Modeling results for each demolition waste type produced by RF.

The predicted values of the RF model by the DW type using the RF algorithm introduced for this study and observed values from the actual survey are compared in Figure 5. The figure shows that the predicted values of the RF models are similar to the pattern of observed values. The graph of all wastes also provides predictions in terms of individual buildings that are similar to the observed values. Therefore, the RF algorithm used in this study for DW generation prediction is expected to exhibit high performance on a small dataset of categorical data.

### 4.3. Discussion

This study developed an AI model for predicting DW generation and proposed an RF model that can make predictions based on each waste type and an entire building, including all wastes based on a small amount of data. 

However, recent studies on DW generation prediction models using AI have used extensive amounts of data. In addition, existing related studies (Kannangara et al. (2018) [27]; Johnson et al. (2017) [46]; Noori et al. (2008) [47]; Azadi et al. (2016) [23]) showed prediction results according to time series. Kannangara et al. (2018) [27] predicted the amount of MSW (Municipal Solid Waste) and paper using neural networks and decision trees. In this study, the R^2^ value of the AI model was 0.35–0.72, and the predictive performance of neural networks was slightly better than that of the decision tree. Johnson et al., (2017) [47] predicted the amount of refuse, paper, and MGP (metal, glass, and plastic) generated monthly by applying the GBM (gradient boosting model), and the prediction performance R^2^ value of the GBM model was 0.428–0.90 It was found that there are differences in predictive performance depending on the type of waste. Azadi et al., (2016) [23] predicted seasonal MSW generation by applying an artificial neural network (ANN) and multiple linear regression (MLR) algorithm. As a result of the study, the ANN (R = 0.86) model was found to be superior to the MLR (R = 0.7) model. However, these studies only dealt with the results of waste generation over time by applying AI algorithms to several waste types. On the other hand, this study presented the results of one RF model for a building unit that included all types of waste, along with the results of the RF models for 11 DW types. In addition, the prediction performance (R = 0.691–0.871, R^2^ = 0.554–0.800) of the model to which the RF algorithm is applied based on a small amount of data can be considered to have excellent prediction performance compared to that of previous studies.

The RF models, shown in this study, are applicable for each type of waste and for a single building. Building demolition work is often conducted in a single building unit; therefore, if a demolition company uses this RF model, it can serve as a useful tool for DW management. In addition, considering the results of the average error between the predicted and observed values described in Section 4.2, the RF model is expected to be used as a powerful management tool even in large-scale dismantling sites.

## 5. Conclusions

In this study, we examined prediction models for DW generation using the RF algorithm. Data preprocessing was conducted on a small dataset to increase the accuracy of the RF model, to which a machine learning algorithm was applied. The input variables were selected by the DW type for modeling. LOOCV was performed to verify the prediction performance of the machine learning model for the small dataset. In total, 11 RF models by the DW type were generated, and one was generated for all DW types. The findings of this study are as follows:

- First, RF is an adequate machine learning algorithm for a small dataset consisting of categorical data. The RF model developed in this study demonstrated a relatively high prediction performance with a high correlation coefficient R of 0.691–0.871 between the values predicted by the models and the observed values.
- Second, the input variables by the DW type deduced from the embedded method of input variable selection, RF-RFE, were applied to the RF model. This implies that, even with a small dataset, an adequate prediction model can be developed. Consequently, we obtained a high prediction performance using three (for mortar) of five (for the rest of the DW types) input variables, apart from concrete (for which six input variables were used).
- Lastly, the results of this study demonstrated a similar pattern for predicted values and observed values from 11 RF models by the DW type and one RF model for a building, including all DW types. In conclusion, this study proposed an RF model that can improve the prediction performance using a small dataset of categorical data.

However, in this study, machine learning was performed using RF because the amount of data was limited. Therefore, future research should extend the range of the data, and comparative analysis should be conducted using various machine learning algorithms to develop a DW management model.

## Figures and Tables

**Table 1:** Characteristics and composition of variables applied to the RF model in this study.

Variables Type			Description
Independent variables type	Nominal variable	Region	Region A is assigned a scale number of 1, and regions B and C are 2 and 3, respectively
		Building use	The scale number is 1 for only residential, and the scale numbers for commercial/residential and only commercial are 2, 3, respectively
		Building structure	Reinforced concrete structure is assigned a scale number of 1, and masonry and wooden structures are 2 and 3, respectively
		Wall material	The scale number for the reinforced concrete wall is 1, the brick wall is 2, the block wall is 3, and the wall made of soil is 4.
		Roofing material	The scale number for the slab is 1, the slab and roofing tile is 2, the roof with asbestos is 3, and the roofing tile is 4.
	Continuous variable	gross floor area (GFA) (m^2^)	Numeric variable
Dependent variable	Continuous variable	Waste generation (kg/m^2^)	Numeric variable

**Table 2:** Results of feature selection produced by RF-recursive feature elimination (RFE). ● Selected Variable Set.

Waste Type	Number of Variables in the Variable Set												Selected Features (the Top 3 Variables Out of 3)
Mortar	1		2		3	●	4		5		6		R, S, A
Concrete	1		2		3		4		5		6	●	RM, R, S, WM, A, U
Block	1		2		3		4		5	●	6		WM, R, S, A, U
Brick	1		2		3		4		5	●	6		WM, RM, R, A, S
Timber	1		2		3		4		5	●	6		R, RM, A, S, U
Slate	1		2		3		4		5	●	6		RM, R, WM, A, S
Roofing tile	1		2		3		4		5	●	6		R, RM, A, WM, S
Plastic	1		2		3		4		5	●	6		R, S, U, RM, WM
Glass	1		2		3		4		5	●	6		R, A, WM, U, S
Metal	1		2		3		4		5	●	6		R, U, RM, WM, S
Soil	1		2		3		4		5	●	6		WM, R, S, A, U

S (structure), R (region), U (building use), A (gross floor area), WM (wall material), RM (roofing material).

**Table 3:** Accuracy assessment of the RF model.

N	RF Model by Waste Type	Statistical Metrics	
		R	R^2^
1	Mortar	0.752	0.561
2	Concrete	0.842	0.707
3	Block	0.840	0.704
4	Brick	0.864	0.745
5	Timber	0.858	0.735
6	Slate	0.814	0.659
7	Roofing tile	0.768	0.583
8	Plastic	0.691	0.568
9	Glass	0.747	0.554
10	Metal	0.871	0.755
11	Soil	0.869	0.800
12	All wastes	0.791	0.615

## Abbreviations

AI	artificial intelligence
C&D	construction and demolition
RF	random forest
DW	demolition waste
GRC	generation rate calculation
GFA	gross floor area
ANN	artificial neural network
SVM	support vector machine
LR	linear regression
DT	decision tree
GA	genetic algorithm
LOOCV	leave-one-out cross-validation
REF	recursive feature elimination
